# Reversion of Types

**Published:** 1910-08

**Authors:** S. O. Young

**Affiliations:** Galveston, Texas


For Texas Medical Journal.

### Reversion of Types.

BY S. O. YOUNG, M. D., GALVESTON, TEXAS.

I am glad to see, from time to time, that the medical profession is beginning to discuss that most interesting question—"heredity." However, such discussion heretofore has been too random and too desultory to accomplish much; the question is too complicated and too intricate to admit of such cursory treatment. One does not realize how difficult it is until one makes a study of the proofs, pro and con, and gives the subject some personal thought and attention.

That the law of heredity prevails among the lower animals is now no longer questioned, but the proof there is all one-sided, for it demonstrates only that bone and muscle can be developed along certain lines and that good or bad qualities in animals may be perpetuated or eliminated. It should be borne in mind, when considering such proof of heredity as this, that it is no proof at all, since it relates to but one phase of the problem—the physical, and neglects altogether the intellectual. When man is considered, the problem becomes complex, for with him both the physical and mental sides must be dealt with. There is but small doubt that by a process of selection a race of giants or a race of pigmies might be developed, but it is extremely doubtful if such favorable results could be obtained if an effort were made to secure a race of poets, philosophers, great historians or authors. The purely animal and the combination of animal and intellect are distinct and separte propositions, and while physical traits might be perpetuated or eliminated in man, just as they are in the lower animals, it is far from probable that the same favorable results would be obtained if the experiment were confined to the intellectual part of man.

If there be truth in the theory, as applied to man, and there must be some, then the subject should receive more careful attention and serious consideration from medical men. Unfortunately, exact and thoroughly reliable data are not readily obtained, and consequently there is at present as much statistical evidence against the theory as there is in its favor. One great drawback is that the period of intelligent observation is too limited. Two, four or half a dozen generations are scarcely enough to afford reliable evidence, one way or the other, for vagrant types, due possibly to causes antedating the series under observation, may develop, thus injecting new equations into the problem.

Every intelligent reader is aware of the fact that there have been families whose records from generation to generation have been remarkable for eminent goodness or eminent wickedness. Here, however, arises the doubt whether this pronounced goodness or badness is due to something inherent in the tissues of the brain or blood corpuscles, or to environment and the personal influence of the elder generation on the younger. Would not the records on either side have been radically changed had the children of the bishop been placed in the hands of the thief and murderer at birth, and those of the criminal been given to the bishop? It is questions such as this that confront one at every step, and since they can never be put to a practical test, of course, they can never be answered.

Two remarkable cases have fallen under my own observation, and I have no doubt that some of my readers whose years have afforded them opportunity to observe can recall one or more of a similar character.

In 1861 there was a young man who was assistant professor of Latin and Greek in one of the leading colleges of the land. His family was one of the oldest and most distinguished in the South. When his State left the Union he resigned his position and enlisted as a private in the Confederate army. He was a good soldier, performing every duty whether on field of battle or in camp. For four years he was faithful and true and never for a moment showed himself to be other than what he was—a gentleman and soldier. After the surrender he sought and found employment as a bookkeeper, and for a number of years held one position. During all that time his conduct was irreproachable. He contributed liberally to the support of his aged father, mother and sisters and as a citizen did his duty as faithfully as he had done when he was a soldier. In 1880, without warning, he quit his desk and left town, without notice to any one, and became a regular tramp. Two years later I saw him at a railroad station in West Texas. He was ragged and dirty, but was fat and apparently well and happy. I have never heard from him since. What was the cause of this sudden reversion of character? Was it the outcropping of some long dead and forgotten ancestor? Certainly there was no one for a generation or two of his immediate ancestors who was responsible for it, for the history of the family, as I have already stated, was one of the most brilliant of the South. There was no love affair, no whisky, no gambling; [illegible] fact, there was no known cause, and, if heredity be ignored, the only explanation that suggests itself is that the devil threw a random shaft and chance guided it to a brilliant mark.

Another case, the reverse of the foregoing, also came under my observation. In 1860 a notorious horse thief was lynched in East Texas for murder. He left three sons and two daughters, all nearly grown. When the war broke out the oldest son entered the Confederate army and was killed during the first year. The second son became a professional gambler, went to Mexico and was killed in a saloon fight. The two girls became prostitutes, and very tough ones, too. The youngest boy sought honest work. He had but the rudiments of an education, but he was ambitious and had talent as a money-maker. After the war he went to another State, where he succeeded in establishing himself in a good business, and is today one of the most esteemed men of the city where he resides.

If ever environment, conditions and adverse influences were combined more strongly against a callow youth than in this case, I would like to hear of it. Was it the return of some God-fearing, praying ancestor, or did some good angel carelessly drop from the ramparts of heaven a golden arrow, tipped with hope and encouragement, which found lodgment in the breast of such an apparently worthless subject as our young man?

Now personally I am inclined to believe in the theory of heredity, but, when I recall such cases are the foregoing, my faith gets shaken and I find myself leaning towards the doctrine of "free-will," absolute personal responsibility and of having "every tub stand on its own bottom."

If heredity could become an established fact, what a delightful thing it would be for a lot of us who are naturally bad. Then some long dead and forgotten ancestor would have to bear the blame for all our sins. Of course, it would make no difference to him and it would be such a comfort to us, when we were caught, to know that our friends and neighbors held some fellow who lived, perhaps, in the seventeenth century, responsible for the whole affair, and felt only pity for us that we should have had the misfortune to have had such a scapegrace among our ancestors.

---

Glycerine dressings covered by rubber tissue are frequently more useful than the ordinary wet dressings in reducing inflammatory swelling and in relieving pain.—*American Journal of Surgery.*

---

In most exploratory laparotomies the seat of disease may be discovered by tracing the attachment of an adherent portion of omentum.—*American Journal of Surgery.*

